# Why to cooperate is better than to compete: brain and personality components

**DOI:** 10.1186/s12868-017-0386-8

**Published:** 2017-09-20

**Authors:** Michela Balconi, Davide Crivelli, Maria Elide Vanutelli

**Affiliations:** 1 Research Unit in Affective and Social Neuroscience, Catholic University of the Sacred Heart, Milan, Italy; 2 Department of Psychology, Catholic University of the Sacred Heart, Largo Gemelli 1, 20123 Milan, Italy

**Keywords:** Cooperation, Competition, Ranking self-perception, EEG, fNIRS, BAS

## Abstract

**Background:**

Cooperation and competition were compared in the present study. Brain correlates (electroencephalography, EEG frequency band, delta, theta, alpha, and beta) and hemodynamic measure of functional near-infrared spectroscopy (fNIRS, O2Hb) were acquired during a joined cooperative (Experiment 1) or competitive (Experiment 2) task. Subjects were required to match each other’s cognitive performance (cooperation) or to make better than others (competition) in terms of accuracy (error rate, ER) and response time (RT). In addition, a personality trait measure (behavioral activation system, BAS) was used to distinguish subjects based on their rewarding attitude. Self-perception of social ranking and real performance were considered in response to subjects’ performance (that was artificially manipulated to show an increasing or decreasing profile during the task).

**Results:**

An increased left prefrontal cortical (PFC) responsiveness was found for subjects who had higher BAS rating in case of both cooperation and competition conditions. Moreover, subjects with higher BAS ratings showed greater frontal left activity during the cooperative task. These subjects also concomitantly perceived an increasing in social ranking and improved their performance.

**Conclusions:**

Present results demonstrated that some trait components (BAS) and cooperative condition induce a positive self-representation in term of ranking and a best way to perform the task, as underlined by self-perception and cognitive outcomes. Indeed the higher BAS trait proved to be related with the representation of higher social ranking and with the perception of improved cognitive outcomes, with also a significant increased left PFC activity in cooperative contexts.

## Background

Cooperation and competition are part of our daily life. When a cooperative or competitive interpersonal task is performed, it may induce different effects that are influenced by the “social” meaning of cooperation or competition and by self-perception in that interpersonal context. When considering social hierarchy, the occurrence of a cognitive task performed together can be accompanied by a modification in our self-representations according to the outcomes. In detail, the perception we built about ourselves is the result of a social analysis on our ranking within a specific situation in which we receive a feedback for our performance. Indeed firstly comparing my own and others’ performance on a specific interpersonal task may (or may not) enhance my rank perception in term of efficacy, taking into account the pre-existing condition. In second instance, the performance related to our cognitive efforts is able to influence self-perception and has an important role in creating a pertinent awareness of our own skills. This process is fundamental for the development of self-improvement in the future [1]. Finally, when we consider social hierarchy perception as a comparison between out performance and others’ skills, it has to be considered that also the behavioral outcomes could be strongly affected, both per se and jointly [1].

But how does this process take place in different interpersonal situations such as cooperation and competition?

Cooperative or competitive performance gained in an interpersonal task substantially implies a process of social comparison together with the explicit assessment of individual performances. Previous work already investigated the relation between self-perception, perceived efficacy, and social hierarchy within a competitive scenario. Findings demonstrated that competition is able to improve individual performances and, contemporarily, to contribute to higher perceptions of the social ranking position based on the behavioral performance [2–4]. However it may implicate a lower sense of in-group partnership and it may make the perception of social membership weaker [5]. In contrast, studies that explored cooperative conditions showed that they are associated with a stronger perceived membership and self-efficacy, a general well-being within the social context, and a reinforced perception of having a high position in the social hierarchy [2, 5–8]. It was also shown that the adoption of a cooperative approach can strengthen interpersonal connection. On the other hand, however, cooperative attitudes could be associated with worse performance than competitive ones [2].

Therefore it is relevant and urgent to distinguish the self-perception of our social efficacy and our position within a social ranking in different interpersonal conditions—namely in competitive or cooperative situations, which produce qualitatively distinct social and psychological dynamics. For example, in our previous study social ranking, as perceived by the individual, was already investigated within a competitive paradigm taking into account also other personality variables [3]. The analyses have been conducted by comparing different experimental conditions with mild or strong social reinforce about subjects’ performance. Nevertheless, no specific studies directly compared the influence of those two contexts (cooperation and competition) in respect to perceivable interpersonal feedbacks. Indeed, it should be noted that cooperation and competition are two basic modes of interpersonal interaction [9, 10]. It has been shown that both cooperative and competitive scenarios imply the adoption of others’ point of view, empathy, and the capacity to adjust our own behaviors according to that of others [11]. However, these subjective capacities are expressed even more during competition which involves the presence of divergent goals [9, 12].

Cooperation and competition also call on different social and cognitive processes. Specifically, it was suggested that empathic and mentalizing attitudes differ somehow between cooperation and competition. Gallagher and Frith [13] suggested that it is important to consider and handle both others’ mental state and reality [14]. This mechanism rely on executive functions and, specifically, on executive inhibition, that is the deliberate suppression of a salient knowledge or response to achieve a personal aim which also comes from inside [15, 16]. Indeed, depending on the interaction modalities (cooperation vs. competition), individuals may either facilitate or hinder others’ goal achievement. When considering competition, the rival’s behavior is much more unpredictable than the cooperative partner. In fact, in this last condition, there are planned and shared expectations about the partner’s behavior, since the goal is common.

Thus, the strong increase in the prefrontal cortex activity—mainly the medial prefrontal cortex—observed during competition may in part mirror higher executive processing demands [9]. Specifically, it was demonstrated that the processing load related to competitive social dynamics are associated with increased brain activation, as indicated by alpha EEG power, across all examined brain regions. As such, competition imposed higher cognitive load. In addition, even the increase in cortico-cortical communication and interconnections was consistent, likely mirroring heightened communication between all strategy planning regions (i.e., prefrontal areas). In contrast, other research demonstrated that one’s own actions are facilitated when actions of the others are more predictable [17, 18]. This is the case in response to cooperation, but the opposite in response to competitive conditions.

Moreover, recent research on the structure and function of neural circuits associated with social perception, social efficacy and social ranking offers preliminary evidence for an anterior neural circuit for those processes related to social cognition. Indeed, it was observed that neural circuits linking limbic, PFC, and striatal structures may be involved in such circuits and related to social responses in their affective, cognitive and behavioral components [19]. Both dorsolateral (DLPFC) and ventrolateral (VLPFC) cortices have proven to be involved during ranking considerations [6, 20, 21]. The activity of these brain areas during social interactions that implicate perception of social performance are likely to be associated with higher-level top down processes over, for example, affective responses when considering social ranking. Such mechanisms are meant to manage appropriate behavioral responses when considering social status. As already suggested by previous evidence, these neural circuits could be recruited to trigger socio-emotional responses and behavioral inhibition [22].

Finally, a main important role is related to motivational aspects and “rewarding” conditions activated by cooperation or competition. In fact, specific brain areas are involved according to task type and rewarding condition. Previously it was found that cooperation furnishes a social motivation and is related to right orbitofrontal activation. Competition, instead, is less socially rewarding, but requires supplementary mentalizing resources. It is associated with higher activity in medial prefrontal areas. Moreover recent research found that motivations and emotions can influence the perception of social position by creating a (more) positive versus negative predisposition in social relationships. Therefore, we supposed that the way people evaluate their position in the social hierarchy partially relies upon some motivational and emotional components, such as the degree to which their actions are well balanced between “approach” attitudes in relation to rewards and absence of punishment, as well as “withdraw” from punishments and absence of reward.

Specifically, it was previously shown that high-BAS individuals (the behavioral activation system, BAS; [23]) show more frequently a dominant attitude within social contexts. This fact is thought to positively influence the subject and his/her representations within the social hierarchy).

On the contrary, high-BIS individuals (behavioral inhibition system, BIS) are often associated with submissive attitudes, with negative consequences on social representations [24]. Generally speaking, the BAS is described as a motivational system which is triggered by rewarding signals and non-punishment, and responsible for approaching and active behavioral patterns.

In addition BAS is generally connected with feelings of dominance and high-BAS people are more sensitive to approach-related emotional contexts, with a favorable and dominant behavior toward the context [25–33]. In previous research, a significant BAS effect was found in distinguishing social hierarchy and social performance [6, 21, 34]. As for the cortical correlates of BIS/BAS, they are deemed as mutually inhibitory and they are lateralized: it has been demonstrated that the left PFC is the cortical location of approach-related motivations and emotions, while the right PFC of withdrawal-related processes [27, 29, 35, 36].

However, as for cortical correlates of cooperation/competition, it remains to be explored if and how the neural activity is differently modulated by competition- or cooperation-induced social evaluations when the cognitive outcome is experimentally manipulated. Indeed no previous studies have manipulated the performance and the ranking position to explore that comparison. Available evidences indicate that during social exchange many brain areas are involved, but it is still to be explored which is the specific contribution of each of them to the agents’ different mind-sets when they compete or cooperate to achieve a shared goal.

To explore the cortical impact of cooperation and competition and the main role of motivational components such as BAS trait, in the present research we monitored electroencephalographic (EEG) and hemodynamic (functional near-infrared spectroscopy, fNIRS) activity in two different experiments (Experiments 1 and 2).

Indeed, firstly, EEG activity may be considered as a good measure of brain responsiveness, and it has often been used to describe distinct responsiveness by the two hemispheres to different emotional and social conditions [27, 28, 37, 38]. Specifically, the hemispheric lateralization model of emotions furnished clear evidences about the significance of the left (more positive valenced stimuli) and right (more negative valences stimuli) hemisphere in correspondence with the alpha band modulation [37]. In addition, EEG modulation was used to demonstrate the lateralized PFC responsiveness related to BAS trait. Indeed, it has been found that a decrease in alpha activity (higher cortical activation) over frontal areas in the left hemisphere typically emerges in response to approach attitude [26, 29, 39–43]. Thus, a hemispheric lateralization was found based on brain oscillations and in concomitance with BIS/BAS distinction. In general, also low-frequency bands can be ascribed to the emotional significance of the stimulus condition. Indeed their modulation was revealed for emotional behavior and in concomitance with high BAS (more left activity) and low-BAS (more right activity). Some studies showed that theta activity is sensitive to emotional stimulation [44, 45], and it was suggested that some specific neurons in the amygdala are related to theta activity during emotional arousal [46–48]. In contrast, few evidences exist on modulations of beta bands in association to the affective significance of a context [49]. For what concerns delta, instead, it has been hypothesized its functional role in signaling novelty within emotional contexts. Also, it could be related to updating processes of affective stimuli in memory [50]. Therefore it seems to respond to attentional salience of the stimulus, more than to its emotional content per se. Focusing on competition, instead, Babiloni et al. [51], in an ecologically valid task simulating a card game, found a increased activity in PFC and anterior cingulated cortex for different frequency ranges for the player who leaded the game, if compared to other players.

Secondly, even if previous work provided functional imaging data associated with social ranking, the temporal features of such processes still need to be addressed. The classical imaging (i.e., functional magnetic resonance, fMRI) measures do not seem to completely describe the real nature of the social inter-personal processes. Thanks to the sudden development of affective and interactive contexts, they require to be studied by imaging techniques that can also provide a sufficient resolution in both temporal and spatial domains and then allow recording event-related hemodynamic responses, such as NIRS [52]. In addition, joint EEG/NIRS techniques permit a simultaneous investigation of electrocortical and hemodynamic features of brain activity during social exchange [53, 54].

Therefore based on our hypotheses, competition versus cooperation may ingenerate different cortical response in PFC based on the underlying more or less rewarding social outcomes. In addition personality components (BAS) was hypothesized to influence perceived hierarchical position—in terms of higher self-perceived abilities for higher BAS—and cognitive performance—namely, improved cognitive outcomes. In other words, the perceived effectiveness of behavior in term of performance during a competitive or cooperative task may be positively modulated by reward mechanisms and consequently these mechanisms may impact on the real cognitive outcomes (improved performance for higher BAS). That is, the improving performance effect should be prominent for high-BAS subjects as a consequence of perceived dominant position and rewarding context, which are clearly positively judged by high-BAS people. Such processes should result in a left lateralized prefrontal activation, as previously discussed about the neural circuits underlying cognitive and social representation. Thus, the need to better explore the role of PFC and its different hemispheric contribution to self-perceived social ranking in combination with personality components [6, 20, 21, 55] is compelling. Some differences were expected between cooperation/competition, with greater left PFC responsiveness for higher BAS in cooperation. Higher BAS subjects in cooperation may more directly beneficiate of this increased PFC activity, due to the increased effect of cooperation on the sense of rewarding by the efficacious joint-actions. Based on previous results, it is likely that an hemispheric “competition” between left and right structures would characterize social hierarchy behavior, with a greater approach attitude and dominance in cooperative condition being associated to a left lateralized pattern. Therefore decreased alpha activity (i.e., increased brain responsiveness) and increased theta (more emotionally and motivationally related) EEG component and increased oxygenated hemoglobin (O2Hb as measured by fNIRS) should emerge for higher-BAS participants compared to higher-BIS participants in the frontal left brain area when they cooperate. In addition, we expected a general increased left more than right brain responsiveness in high-BAS when they cooperate.

To summarize, the three compartments of social ranking perception, personality components and cognitive performance should have a common trend, since we expected a correlated increased self-perception of social ranking and a better performance in relation to higher-BAS, with a subsequent higher activation over left frontal areas.

## Experiment 1

### Methods

#### Subjects

Twenty-two undergraduate students (M = 22.13, SD = 1.98; male = 12) took part in the experiment. All participants were right-handed with normal or corrected-to-normal visual acuity. Exclusion criteria consisted in the presence of a psychopathological history for the subjects and immediate family. In addition, State-Trait-Anxiety-Inventory (STAI, [56]) and Beck Depression Inventory (BDI-II, [57]) were administered after the experimental session. No neurological or psychiatric pathologies were observed. No payment was provided for subjects’ performance. They gave informed written consent to participate in the study. The research was approved by the local ethics committee of the Department of Psychology, Catholic University of Milan.

#### Procedure

Participants were accommodated in a moderately lit room in front of a monitor screen positioned at around 60 cm from their eyes. They were required to complete a simple task on sustained selective attention (modified from the original version: [21]). Subjects were informed that some measures about their attentive performance would have been used to evaluate personal skills and, to improve their motivation, that these indices are usually adopted to assess potential career success and teamwork capacities. In addition, the cooperative goal of the task was underlined. Participants were also informed that the scorings were calculated according to their ability to produce synchronized responses with their partner, in term of accuracy (% of correct responses) and response times (RTs). They were positioned side-by-side but they could not interact each other since they were divided by a black screen.

**Fig. 1:**
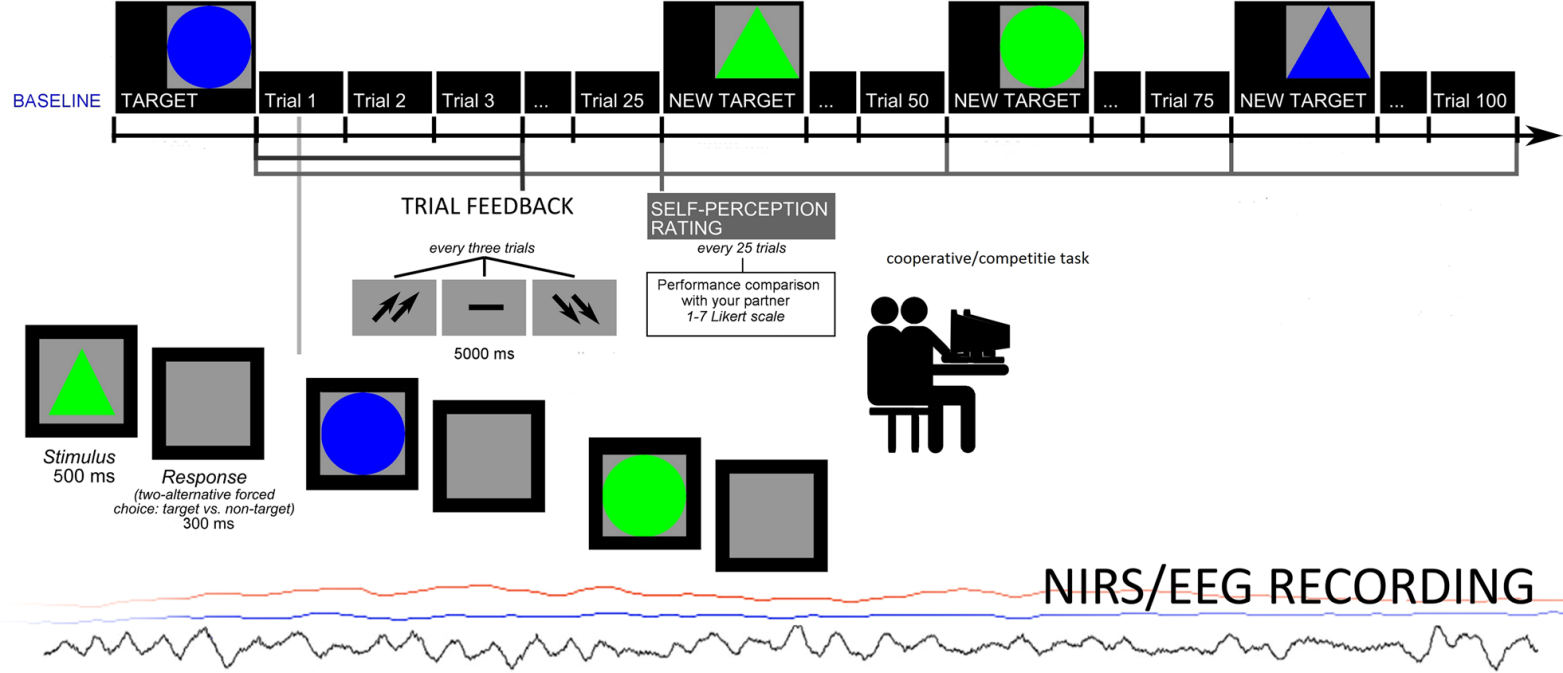
Experimental procedure which represents setting, task and EEG and O2Hb measure for both cooperation (Experiment 1) and competition (Experiment 2)

The instruction was to choose target stimuli between non-targets. Stimuli consisted in geometric shapes and were arranged according to four shape/color combinations: triangles or circles, blue or green. The target remained on the video to be memorized and then all the experimental stimuli were displayed one after another. The target stimulus changed every 25 trials. Subjects were required to make a two-alternative forced-choice by pressing a left/right button. Stimuli were displayed for 500 ms, and separated by a 300 ms inter-stimulus interval (ISI). After each trial, constituted by three stimuli, a feedback was presented on the screen as two up-arrows (high cooperation score), a dash (mean performance), or two down-arrows (low cooperation score). The feedback was presented for 5000 ms, and it was preceded and followed by 5000 ms blanks. The task was composed by 8 blocks (for a total of 200 trials) (Fig. 1). Halfway, subjects were provided with a general evaluation of their cooperative performance. Both feedbacks and the intermediate evaluation were fixed by the experimenter, and couples were told they had a good cooperative (synchronicity) score with 89% about speed synchrony, and 94% about accuracy synchrony. They were also asked to keep their performance level during the second half of the task. Across the experiment, after a preliminary phase with a mean performance, participants were continually reinforced about their proficient cooperation scores by presenting the positive feedbacks (up-arrows) in 70% of cases, and the neutral or negative feedbacks (dash or the down-arrows) in 30% of cases. Moreover, after answering all the stimuli in each block (25 trials), participants were asked to complete a self-evaluation scale about their performance and ranking by using a seven-point Likert scale (1 = very low ranking due to performance, to 7 = very high ranking). Subjects reported to be strongly engaged in the hierarchical situation (94% told to be strongly engaged), as assessed by post-session questionnaire data. Participants were also required to state their trust level about the provided feedbacks on the performance (which showed high trust, 94%), the relevance of the game for social status (97%), and the potentially perceived upgrading of ranking position during the experiment (92%).

#### BAS scores

BAS scores were calculated for each subject (Italian version [58] of Carver and White [59] Questionnaire). The questionnaire included 24 items (20 score-items and 4 fillers, measured on a four-point Likert scale), and two total scores for BIS (range = 7–28; items 7) and BAS (range = 13–52; items 13). BAS includes three different subscales (Reward, 5 items, Drive, 4 items, and Fun Seeking, 4 items). Two total scores (BIS and BAS total) and three BAS subscale scores have been calculated. The mean values and standard deviations for each scale were respectively: BAS: 48.13 (3.89); Reward: 23.70 (2.64); Drive: 13.88 (1.97); Fun Seeking: 13.54 (2.87). Cronbach’s alpha was calculated for BAS (.87) and for each BAS subscale (Reward .88; Drive .88, and Fun Seeking .91). Since BIS and BAS were orthogonally distributed and systematically participants higher in BAS were lower in BIS, BIS was not used in this phase of research. One subject was not considered in final analyses since he showed a mixed-profile (both high-BAS and high-BIS score). The questionnaire was given to the subjects after completing the experimental phase.

#### EEG recording and analysis

EEG recordings were conducted with two 16-channel EEG-systems (V-AMP: Brain Products, München. Truscan: Deymed Diagnostic, Hronov). An ElectroCap with Ag/AgCl electrodes was applied to record EEGs from active sites on the scalp referred to the earlobes (10/5 international system; [60]). Data were acquired using a sampling rate of 500 Hz, with a frequency band of .01–40 Hz. An off-line common average reference was computed later to attenuate the problems related with the signal-to-noise ratio [61]. One EOG electrode was positioned on the outer canthi to identify eye movements. The impedance of the recording electrodes was supervised for each subject before beginning data collection and was always below 5 kΩ. The signal was visually inspected, and those portions of data that contained artifacts were removed to increase specificity. Blinks were also visually checked. Ocular artifacts (eye movements and blinks) were corrected by using an eye-movement correction algorithm that applies a regression analysis together with artifact averaging [62]. After performing EOG correction and visual inspection, only artifact-free trials were included (rejected epochs, 2%).

**Fig. 2:**
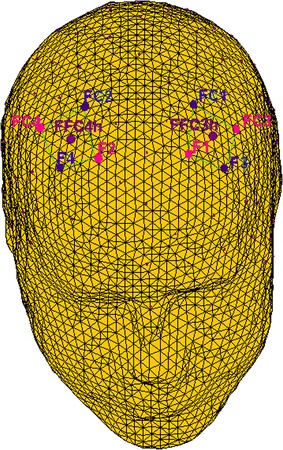
The location NIRS channels. NIRS: The emitters were placed on positions FC3–FC4 and F1–F2, while detectors were placed on FC1–FC2 and F3–F4

The digital EEG data were bandpass filtered in the frequency bands: delta (.5–4), theta (4–8), alpha (8–12 Hz), beta (14–20) (band-pass filtering 96 dB/octave rolloff, warm-up filter left and right to 100 ms). To obtain a signal proportion to the power of the EEG frequency band, the filtered signal samples (epoch 1000 ms) were squared [63]. An average absolute power value for each experimental condition was calculated, as well as of the pre-experimental absolute power (−200 ms), that was used to determine the individual power without experimental stimulation. For the statistical analyses only left and right frontal (FFC3h, FFC4h) power activity for each frequency band was considered [64] (Fig. 2).

#### fNIRS recording and analysis

fNIRS measurements were performed with the NIRScout System (NIRx Medical Technologies, LLC., Los Angeles, California) using an 8-channel arrangement of optodes (4 light sources/emitters and 4 detectors) positioned over the prefrontal area. Optodes were placed on frontal and fronto-central sites (Sources: FC3–FC4 and F1–F2; Detectors: FC1–FC2 and F3–F4) (Fig. 2). Emitter–detector distance was kept at 30 mm for contiguous optodes. Also, a near-infrared light of two wavelengths (760 and 850 nm) was used. NIRS optodes were applied to the subject’s head using a NIRS-EEG compatible cap, by considering the international 10/5 system.

For data acquisition, NIRStar Acquisition Software was used to detect changes in the concentration of oxygenated hemoglobin (O2Hb) and deoxygenated hemoglobin (HHb). A starting baseline (120 s) was also recorded. Signals obtained from the eight channels were acquired with a sampling rate of 6.25 Hz, and analyzed and transformed according to their wavelength and location. The result of this procedure consisted in values for the changes in the concentration of oxygenated and deoxygenated hemoglobin for each channel. Hemoglobin quantity is scaled in mmol ∗ mm, implying that all concentration changes depend on the path length of the NIR light in the brain.

The raw data of O2Hb and HHb from each channel were digitally band-pass filtered at .01–.3 Hz. Successively, the mean concentration within each subject was calculated by averaging data across the trials from the feedback onset for 5 s. According to the mean concentrations in the time series, we computed the effect size in every condition for each channel within a subject. The effect sizes (Cohen’s d) were calculated as the difference of the means of the baseline and trial divided by the standard deviation (SD) of the baseline: d = (m_1_ − m_2_)/s. Accordingly, m_1_ and m_2_ are the mean concentration values during the baseline and trial, and s means the SD of the baseline. The mean concentration value of 5 s immediately before the trial was used as event-related baseline. Then, the effect sizes obtained from the eight channels were averaged in a way to increase the signal-to-noise ratio. Although NIRS raw data were originally relative values and could not be directly compared across subjects or channels, this procedure that normalized data allowed averaging regardless of the unit [65–67]. In fact, the effect size is not affected by differential pathlength factor (DPF) [66].

### Results

Four different levels of analyses were applied by considering to behavioral (error rates, ERs; response times, RTs; ranking self-perception) and neurophysiological (frequency ranges: delta, theta, alpha and beta; O2Hb modulation) measures. Behavioral measures have been entered into repeated measure ANCOVAs which included the covariate factor BAS (two levels, high- vs. low), while analyses applied to each frequency band and O2Hb measures included both BAS as covariate and Lateralization (Lat, two levels, left vs. right) as independent factor.

RTs have been calculated from the stimulus presentation, and ERs, the number of wrong answers, were computed as a percentage within each experimental condition. Accordingly, higher scores reflect worse responses: longer and inaccurate. For what concerns perceived self-efficacy, ranking score was considered (for this variable see “Procedure”). Band modulation and O2Hb and HHb were calculated as the mean values during the performance. For all of the ANCOVA analyses, the degrees of freedom were adjusted by using Greenhouse–Geisser epsilon if needed. For significant interaction effects, paired contrast analyses were also conducted. Bonferroni correction was adopted for multiple comparisons.

As last step, different sets of correlations were run between behavioral performance (ER; RTs), perceived self-efficacy, O2Hb, and the four frequency bands.

#### ANCOVA

##### ERs

**Fig. 3:**
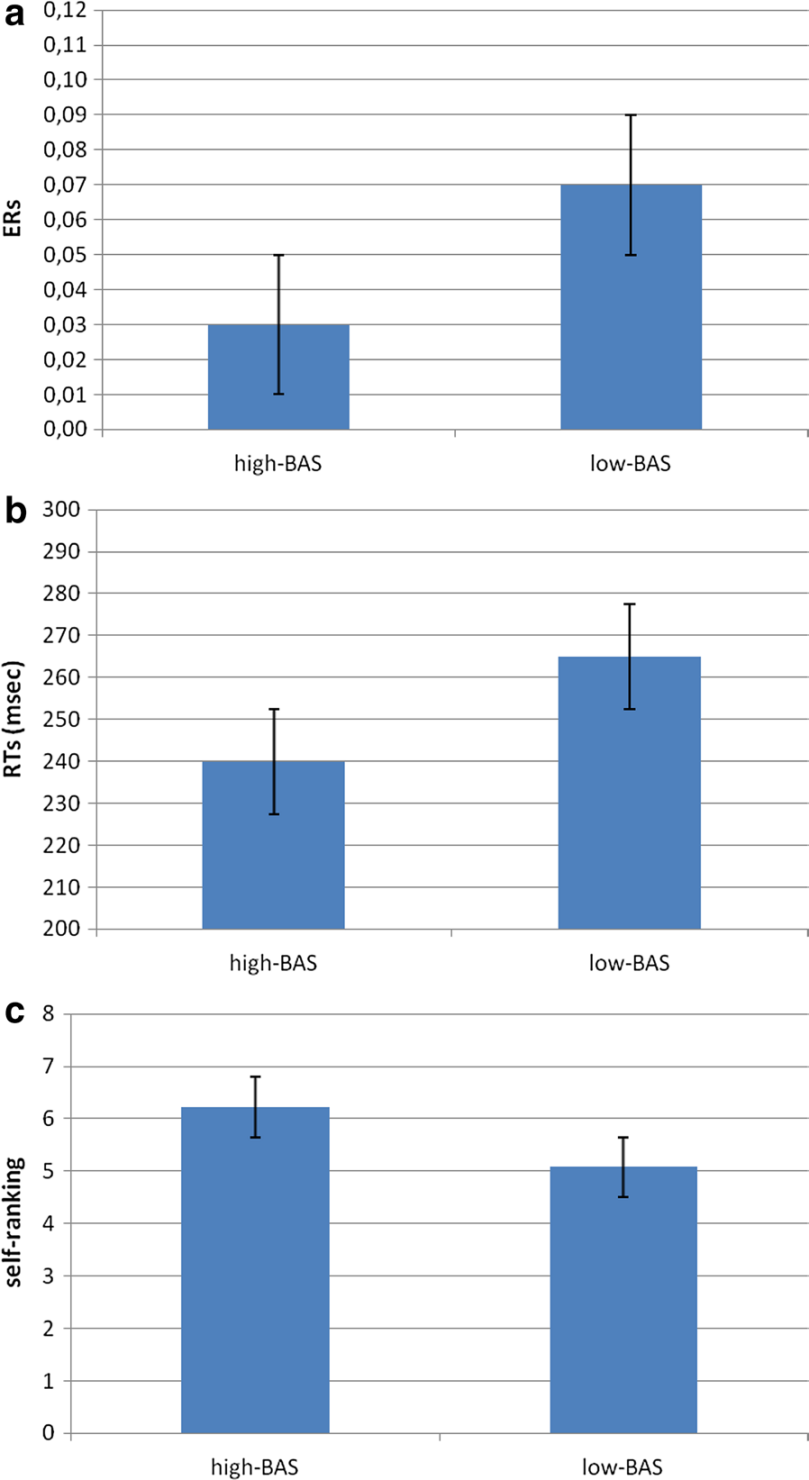
**a** ERs, **b** RTs and **c** self-perception modulation as a function of BAS

Results indicated a significant effect for BAS (*F* [1, 21] = 8.23, *p* ≤ .001, *η* ^2^ = .37). Indeed high-BAS rating showed a decreased ER compared to low-BAS rating. In order to display the high-BAS versus low-BAS rating differences, Fig. 3a and the following ones represent the high-BAS and low-BAS subjects based on two cut-offs, more than 50 (mean + 1 SD, N = 10) for high, and less than 44 (mean − 1 SD, N = 12) for low-BAS subjects.

##### RTs

ANOVA indicated significant main effects for BAS (*F* [1, 21] = 6.98, *p* ≤ .001, *η* ^2^ = .33), with a general decreased RTs for high-BAS compared to low-BAS rating (Fig. 3b).

#### Self-ranking

About the evaluation of the ranking position in term of performance, ANCOVA indicated significant effect for BAS (*F* [1, 21] = 8.55, *p* ≤ .001, *η* ^2^ = .38). Indeed high-BAS rating showed higher ranking perception than low-BAS rating (Fig. 3c).

#### Frequency band analysis

**Fig. 4:**
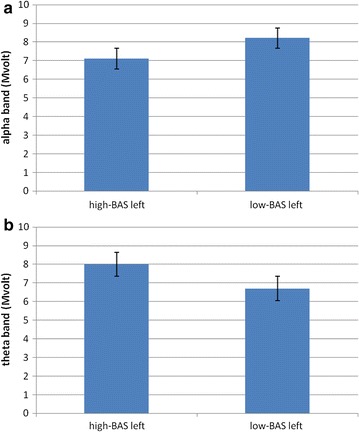
Alpha (**a**) and theta (**b**) variation as a function of BAS within the left hemisphere. High-BAS showed increased left response compared to low-BAS

About delta and beta no main or interaction effect was significant at the analysis. With regard to theta, ANCOVA indicated significant effects for Lat (*F* [1, 21] = 7.60, *p* ≤ .001, *η* ^2^ = .36) and Lat × BAS (*F* [1, 21] = 8.04, *p* ≤ .001, *η* ^2^ = .39). The significant post hoc effects, that we reported, showed increased left theta activity for high-BAS compared to low-BAS rating (*F* [1, 21] = 6.78, *p* ≤ .001, *η* ^2^ = .33), whereas no significant differences were found within the right hemisphere based on high-low-BAS (*F* [1, 21] = 2.01, *p* = .23, *η* ^2^ = .11). A higher left than right responsiveness was also revealed for high-BAS rating (*F* [1, 21] = 7.11, *p* ≤ .001, *η* ^2^ = .33). About alpha ANCOVA indicated significant main effects for Lat × BAS (*F* [1, 21] = 7.76, *p* ≤ .001, *η* ^2^ = .35), with decreased left alpha activity (increased brain response) for high-BAS rating compared to low-BAS rating (*F* [1, 21] = 7.90, *p* ≤ .001, *η* ^2^ = .36); whereas no significant differences were found within the right hemisphere (*F* [1, 21] = 1.92, *p* = .32, *η* ^2^ = .22). In addition high-BAS rating showed decreased alpha in the left than in the right side (*F* [1, 21] = 8.04, *p* ≤ .001, *η* ^2^ = .36) (Fig. 4a, b).

#### fNIRS

The statistical analyses were applied to d dependent measure for O2Hb and HHb-concentration. The analysis on HHb did not reveal significant effect and, for this reason, we reported only results for O2Hb-values. D dependent measures were fed to Lat factor and BAS covariate repeated measure ANCOVA. For Lat factor the data were averaged over the left (F1–F3, F1–FC1, FC3–FC1, FC3–F3) and the right (F2–F4, F2–FC2, FC4–FC2, FC4–F4) channels.

**Fig. 5:**
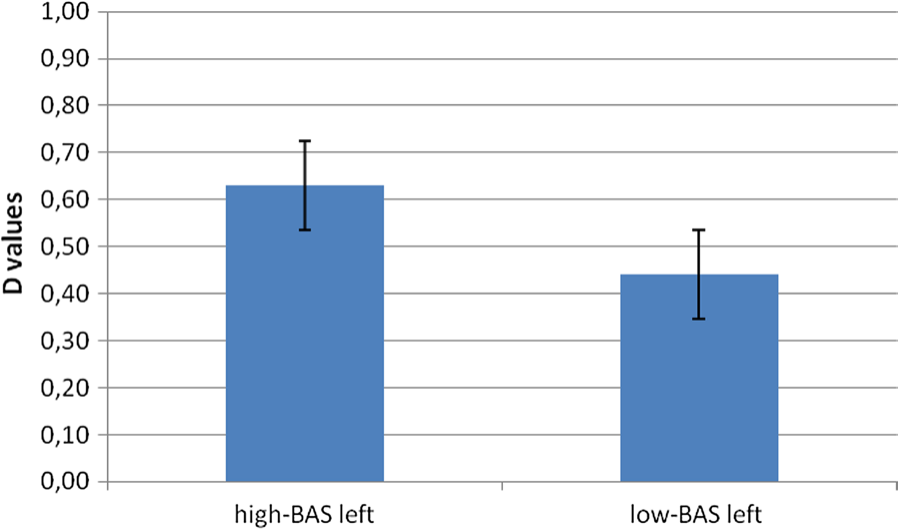
O2Hb modulation (D values) as a function of BAS. High-BAS showed increased left-lateralized response compared to low-BAS

As shown, Lat (*F* [1, 21] = 7.98, *p* ≤ .001, *η* ^2^ = .35) and Lat × BAS effects were significant (*F* [1, 21] = 9.13, *p* ≤ .001, *η* ^2^ = .39). About the main effect, it was observed a general increased left activity and a specific left increased response for high-BAS scoring (*F* [1, 21] = 9.78, *p* ≤ .001, *η* ^2^ = .40). Moreover, about the simple effects, it was observed an increased response for high-BAS scoring within the left more than the right hemisphere (*F* [1, 21] = 7.78, *p* ≤ .001, *η* ^2^ = .35) (Fig. 5).

#### Correlation analysis

A series of correlation analysis was applied to cognitive performance (ERs; RTs), self-perception, D, and EEG modulation. Pearson correlation coefficients were calculated between them. BAS revealed significant positive correlation with self-ranking (r^2^ = .498, *p* ≤ .001) and performance (RTs) (r^2^ = .509, *p* ≤ .001). In addition BAS was inversely correlated with alpha within the left hemisphere (r^2^ = −.399, *p* ≤ .001) (increased left brain activity in concomitance with higher BAS), and directly correlated with theta (r^2^ = .543, *p* ≤ .001) and D modulation (r^2^ = .467, *p* ≤ .001). In addition self-ranking was inversely correlated with RTs (r^2^ = −.464, *p* ≤ .001) and directly correlated with theta (r^2^ = .578, *p* ≤ .001) (increased left activity in concomitance with higher self-ranking) and O2Hb (r^2^ = .525, *p* ≤ .001). Finally O2Hb and theta band proved to be correlated (r^2^ = .515, *p* ≤ .001).

## Experiment 2

### Methods

#### Subjects

Thirty undergraduate students (M = 22.38, SD = 2.55; male = 13) took part in the experiment. The same selection criteria adopted for Experiment 1 were used in the Experiment 2.

#### Procedure

The same procedure of Experiment 1 was adopted, with a specific variation in term of the nature of the task. Indeed in Experiment 2 the competitive task was stressed: participants were told that the scoring was based on the capacity to beat the partner, in term of accuracy and speed. Also in this case a general (fake) evaluation was presented halfway. Subjects were told that their performance was “well above” or “well below” than their rival’s one and were required to maintain their outcomes (for winners) or to improve it (for losers) during the second half of the experiment (“The measures recorded till now reveal that”: for winners: “your performance is very good. Your response profile is well superior to your competitor’s one. If you want to win, keep going like this in the following part”; for losers: “your performance is really poor. Your response profile is well inferior to your competitor’s one. If you want to win, you’ll have to improve your performance in the following part”). During the task, the trial feedbacks constantly reinforced participants about their good performance (in the case of winners) by presenting the up-arrows in 70% of cases, while the dash or the down-arrows only in 30% of cases (mainly at the beginning of the task) to make the task more credible and plausible. The opposite arrangement was proposed in the case of losers.

As shown by post-session questionnaire, participants were strongly engaged in the hierarchical situation (92%), with high trust in the feedback (96%), with a good perception of relevance of the task for social status (94%), and with a perceived improved ranking position during the task (93%).

#### BAS score

The total scores for each scale were respectively: BAS: M = 47.90 (SD = 3.91); Reward: M = 23.76 (SD = 2.03); Drive: M = 12.98 (SD = 1.15); Fun Seeking: M = 13.09 (SD = 1.23). Cronbach’s alpha was calculated for BAS (.98) and for each BAS subscale (Reward = .88; Drive = .90, and Fun Seeking = .91).

### Results

#### ANOVA

##### ERs

**Fig. 6:**
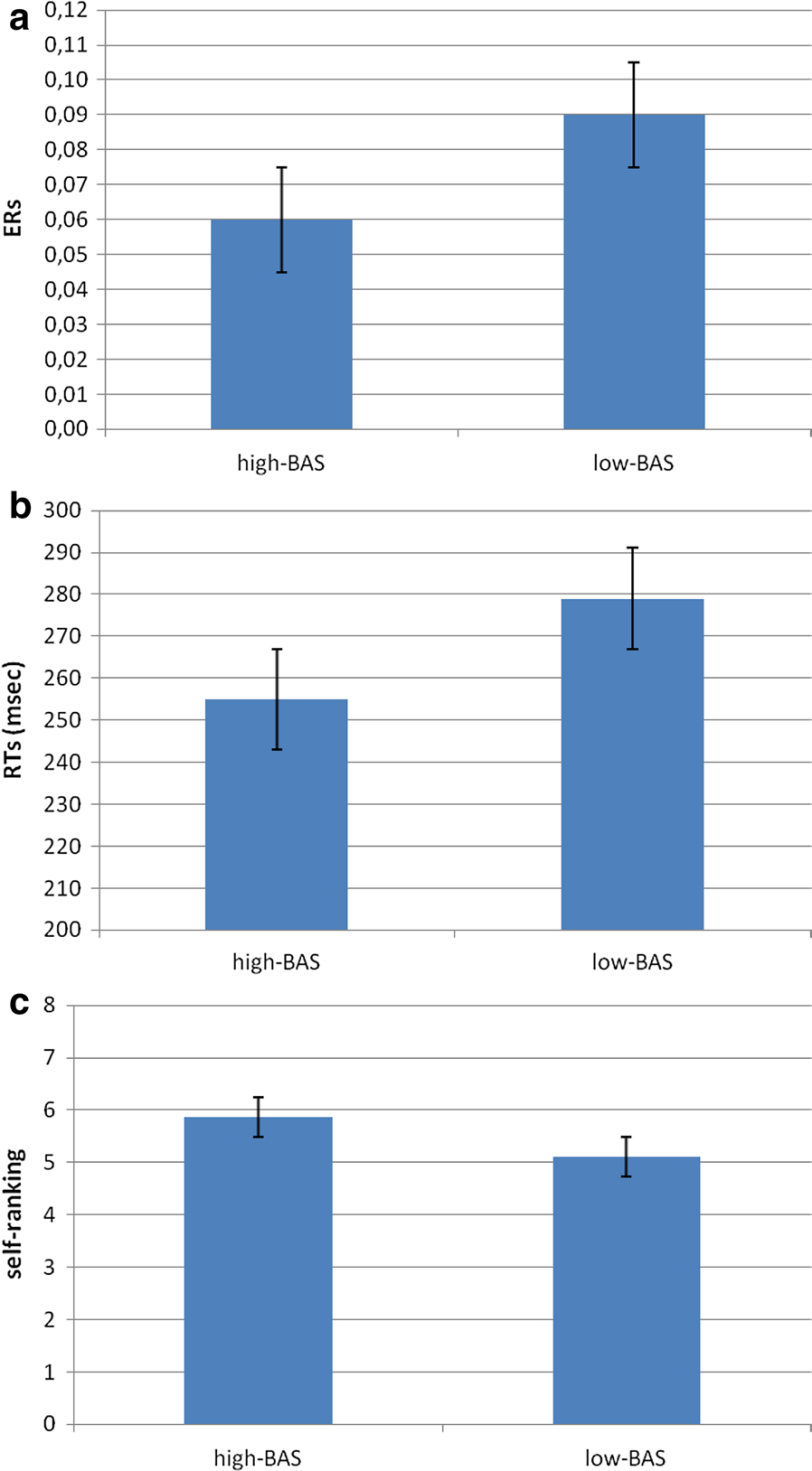
**a** ERs, **b** RTs and **c** self-perception modulation as a function of BAS

ANOVA indicated significant effect for BAS (*F* [1, 29] = 6.78, *p* ≤ .001, *η* ^2^ = .32). High-BAS showed a decreased ER than low-BAS (Fig. 6a). In order to display the high-BAS versus low-BAS rating differences, the present figure, and the following figures, represents the high-BAS and low-BAS ratings based on two cut-offs, i.e., includes subjects with high BAS scoring more than 49 (mean + 1 SD, N = 13); and low-BAS scoring less than 44 (mean − 1 SD, N = 17).

##### RTs

ANOVA indicated significant main effect for BAS (*F* [1, 29] = 8.03, *p* ≤ .001, *η* ^2^ = .38), with decreased RTs for high-BAS than low-BAS scoring (Fig. 6b).

#### Self-ranking

About ranking position, ANOVA indicated significant effect for BAS (*F* [1, 29] = 7.55, *p* ≤ .001, *η* ^2^ = .37). Indeed high-BAS scoring showed higher ranking perception than low-BAS scoring (Fig. 6c).

#### Frequency band analysis

**Fig. 7:**
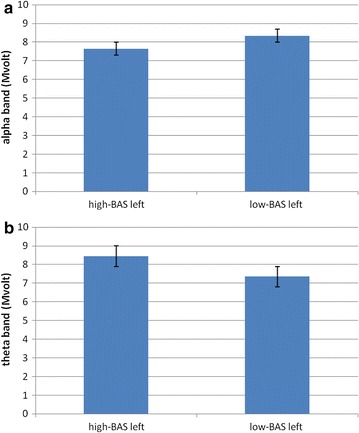
Alpha (**a**) and theta (**b**) variation as a function of BAS within the left hemisphere. High-BAS showed increased left response compared to low-BAS

As for delta and beta bands no main or interaction effect was significant at the analysis. With regard to theta, ANOVA revealed significant main effect for Lat (*F* [1, 29] = 7.65, *p* ≤ .001, *η* ^2^ = .35), with increase left than right activity; and significant interaction effect for Lat × BAS (*F* [1, 29] = 8.55, *p* ≤ .001, *η* ^2^ = .38), with increased left theta activity for high-BAS scoring compared to low-BAS scoring (*F* [1, 29] = 7.32, *p* ≤ .001, *η* ^2^ = .32). A more left than right responsiveness was also revealed for high-BAS scoring (*F* [1, 29] = 7.43, *p* ≤ .001, *η* ^2^ = .36). As for alpha activity, the analysis indicated significant interaction effect Lat × BAS (*F* [1, 29] = 7.88, *p* ≤ .001, *η* ^2^ = .37), with decreased left alpha activity (increased brain response) for high-BAS scoring compared to low-BAS scoring (*F* [1, 29] = 9.77, *p* ≤ .001, *η* ^2^ = .40); whereas no significant differences were found within the right hemisphere (*F* [1, 29] = 1.12, *p* = .31, *η* ^2^ = .21). In addition high-BAS scoring showed decreased alpha more in the left side than in the right side (*F* [1, 29] = 9.65, *p* ≤ .001, *η* ^2^ = .40) (Fig. 7a, b).

#### fNIRS

**Fig. 8:**
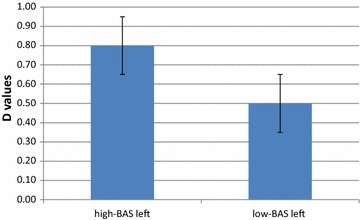
O2Hb modulation as a function of BAS. High-BAS showed increased left-lateralized response compared to low-BAS

As shown by ANOVA, Lat (*F* [1, 29] = 7.65, *p* ≤ .001, *η* ^2^ = .37) and Lat × BAS were significant (*F* [1, 29] = 7.89, *p* ≤ .001, *η* ^2^ = .35), with a general increased left more than right brain activity. Moreover, reporting the significant simple effect, it was observed an increased response for high-BAS scoring within the left more than the right hemisphere (*F* [1, 29] = 7.83, *p* ≤ .001, *η* ^2^ = .36). In addition left PFC was more responsive for high-BAS than low-BAS scoring (*F* [1, 29] = 9.20, *p* ≤ .001, *η* ^2^ = .40) (Fig. 8).

#### Correlation analysis

Pearson correlation coefficients were calculated between ERs, RTs, self-perception, O2Hb and EEG. BAS revealed significant positive correlation with self-ranking (r^2^ = .455, *p* ≤ .001) and inverse correlation with performance (RTs) (r^2^ = −.593, *p* ≤ .001). In addition BAS was inversely correlated with alpha within the left hemisphere (r^2^ = −.496, *p* ≤ .001) (increased left brain activity in concomitance with higher BAS), and directly correlated with theta (r^2^ = .560, *p* ≤ .001) and O2Hb modulation (r^2^ = .499, *p* ≤ .001). In addition self-ranking was inversely correlated with RTs (r^2^ = −.432, *p* ≤ .001), alpha modulation (r^2^ = −.439, *p* ≤ .001), and directly correlated with theta and O2Hb (r^2^ = .555, *p* ≤ .001). Finally O2Hb and theta band were significantly correlated (r^2^ = .570, *p* ≤ .001).

#### Comparison between Experiment 1 and Experiment 2

A direct comparison between the two experiments was conducted for the dependent measures of ERs, RTs, self-ranking, frequency band and O2Hb. The independent factor experiment (Experiments 1 vs. 2) was added to previous statistical design (see Experiments 1 and 2 for the statistical design). We reported only the significant effects where Exp factor was significant, to synthesize the main results.

##### ERs

ANCOVA indicated significant effect for Exp (*F* [1, 51] = 7.85, *p* ≤ .001, *η* ^2^ = .36) and BAS × Exp (*F* [1, 51] = 6.85, *p* ≤ .001, *η* ^2^ = .33). Firstly, Exp 1 showed decreased ERs than Exp 2 (*F* [1, 51] = 7.11, *p* ≤ .001, *η* ^2^ = .34). Secondly in Exp 1 high-BAS scoring revealed reduced ERs than in Exp 2 (*F* [1, 51] = 8.79, *p* ≤ .001, *η* ^2^ = .39).

##### RTs

ANCOVA indicated significant no significant effect which included the Experiment factor.

#### Self-ranking

About the evaluation of their ranking position, ANCOVA indicated significant effect for Exp (*F* [1, 51] = 6.90, *p* ≤ .001, *η* ^2^ = .34) and BAS × Exp (*F* [1, 51] = 7.99, *p* ≤ .001, *η* ^2^ = .36). Indeed in Exp 1 self-perceived ranking was evaluated higher than in Exp 2 (*F* [1, 51] = 6.50, *p* ≤ .001, *η* ^2^ = .30). Finally high-BAS scoring showed increased self-ranking perception in Exp 1 than in Exp 2 (*F* [1, 51] = 7.41, *p* ≤ .001, *η* ^2^ = .34).

#### Frequency band

While no main or interaction effects proved to be significant for alpha, delta and beta data, ANCOVA revealed significant effects for theta bands. About theta Lat × BAS × Exp was significant (*F* [1, 51] = 7.59, *p* ≤ .001, *η* ^2^ = .35): a more left responsiveness was revealed for high-BAS scoring in Exp 1 than in Exp 2 (*F* [1, 51] = 7.27, *p* ≤ .001, *η* ^2^ = .35). In addition high-BAS compared to low-BAS scoring showed increased theta in Exp 1 (*F* [1, 51] = 7.10, *p* ≤ .001, *η* ^2^ = .35).

#### fNIRS

As shown by ANCOVA, Lat × BAS × Exp (*F* [1, 51] = 7.56, *p* ≤ .001, *η* ^2^ = .37) interaction effects were significant. It was observed an increased response for high-BAS scoring within the left more in Exp 1 than Exp 2 (*F* [1, 51] = 7.61, *p* ≤ .001, *η* ^2^ = .38).

### Discussion

The present research intended to explore the brain correlates and the effect of personality components (BAS) in social ranking perception and cognitive performance during a task which included a cooperative (Experiment 1) or a competitive joint-action (Experiment 2). Specifically EEG (brain oscillations) and fNIRS hemodynamic brain activity (O2Hb) were considered in subject showing high- and low-BAS profile, to elucidate how this motivational trait component may affect subjects in formulating self-representation (ranking position) and self-improvement (cognitive performance) in an interpersonal cooperative and competitive context.

A first main effect elucidated by the present research was related to the implication of PFC during the cooperative and competitive task. Indeed in both Experiments 1 and 2 the electrodes positioned over the PFC showed to be significantly modulated in response to cooperation and competition, with a significant increased activity mainly within the left side. In addition, both EEG and fNIRS measures showed a significant and increased lateralization effect in conjunction with BAS score. Such finding replicated previous results which suggested a similar role for some prefrontal areas (such as the VMPFC) in response to status perception [68]. Recent studies investigating the effect of interpersonal situations and reciprocal strategies, when interacting with cooperative and non-cooperative human partners, highlighted the presence of specific activations in the DLPFC [3, 4, 69] and increased activity in the superior temporal sulcus when cooperating positively with a computer [70]. Moreover, using the EEG hyper-scanning technique it was reported a specific involvement of this area when interacting during Prisoner’s Dilemma games [71].

As for the specific contribution of some frequency bands (mainly alpha and theta more than delta and beta) that we found to be relevant to explain the cortical activation, we may suggest that from one hand alpha may function as an index of brain lateralized activation. Specifically, it has been proven that a decrease in alpha power could indicate increased brain activity and a differential responsiveness by the two hemispheres in relation to specific cognitive or affective tasks [29, 72]. From the other hand, theta was previously considered as a specific index of motivational and emotional aspect, as well as of salience of the task and of the subjects’ engagement in the task itself.

Indeed, previous work highlighted that event-related theta activity signal sustained visual stimulation with affective content [44, 46, 53, 73] when coordinated responses are needed to guarantee alertness and readiness. Specifically, it was shown that, when dealing with attentive functions, theta activity is mainly localized over frontal sites. Generators reconstruction analyses also showed how such activation could be produced within cortico-hippocampal and frontolimbic networks [47, 49]. Also, it has been shown that theta oscillations are involved in memory and emotional regulation [44] and, more recently, Kawasaki and Yamaguchi [74] found that frontal theta activity increased during interval periods while waiting for a monetary reward. In some studies theta power has also been shown to increase when goal conflicts are experienced [75–77]. However in the present context we may suggest that theta may preferentially functions as a marker of the salience of the task [46, 47, 49] and of the positivity of the interpersonal outcomes, as indicated by its sensitivity to positive feedback to the joint-action. In fact, it has been suggested that EEG frequency within theta range could be related to implicit processes within social cognition [78].

A second interesting finding consists in the recruitment of the PFC that was modulated as a function of both BAS trait and the cooperative/competitive nature of the task. This effect emerged in the modulation of EEG activity recorded over prefrontal sites and was confirmed by optical imaging analyses (fNIRS).

Indeed, in addition to this general enhanced left PFC activity, a specific lateralization pattern was found, with increased left activation with respect to the right one, mainly for higher-BAS and mainly in the case of cooperative task. Firstly, the “left hemisphere effect” in integration with the “approach attitude” was found to be leading to explain our results. This is in line with previous studies, which reported that high-BAS profiles were more likely to relate to the dominant and “proactive” attitudes in situations that were shown to induce a positive and rewarding effect [24]. Specifically, it was also demonstrated that, considering resting intracortical activity during social emotionally salient task, participants with increased left versus right DLPFC activity also displayed also showed more frequently adaptive, dominant, and approach-related responses [79].

Secondly, high-BAS scoring showed significantly greater PFC activation in case of cooperation than of competition and this brain response was related to improved performance and increased self-perceived ranking position, as was highlighted by both EEG (alpha decreasing and theta increasing) and fNIRS (O2Hb increased values). As underlined by previous data, we may explain these results taking into account evidences from evolutionary and developmental psychology, which underline that cooperating is more gratifying than competing from a social point of view since cooperation may be represented as a source of positive social feedback on the joint performance towards the common objective, in addition to the intra-subjective positive feedback as in competition [80]. Previous neuroimaging work [81] suggested the involvement of the medial orbitofrontal and anterior frontal cortex in after positive feedback and outcomes. In fact, the presence of a performance-related feedback was manipulated in different planning and guessing tasks. Another work with fMRI has revealed that the orbitofrontal cortex seems to process rewarding values after comparison, and not absolute gratifying stimuli [82]. Considering the neural substrates, it has been previously shown that the PFC is implicated when controlling finalized behaviors [83]. Moreover, the left orbitofrontal areas seem specifically related to rewarding conditions, as affirmed by the approach-withdrawal theory [84–86]. We propose that results from the current study highlight how the rewarding meaning comes from the psychological gratification to achieve a shared objective by interacting with another mate. This suggestion would also be supported by the idea that social sharing can be rewarding per se [87] and that this rewarding condition was more related to cooperation than competition, it being probably due to the social relevance of cooperation for the inter-subjective survival and the reciprocal integration during an interactive social exchange.

As for the cognitive performance it was also found a relevant direct relationship between the brain activity within the left PFC, the cognitive behavior and the social representation for both cooperation and competition, as shown by the correlation analyses. The effect related to the cortical “unbalance” over the left hemisphere in response to cooperation and competition was also associated with an increased sense of social efficacy and a concomitant better performance (decreased ERs and RTs) could support a possible connection between left-sided prefrontal activation, social hierarchy representation, and behavioral change.

The cortical lateralization could support the presence of a significant over-activation of left anterior regions and, in parallel, the role of such areas in managing the subjective perception to be upper in ranking. To verify this hypothesis, previous work highlighted that the subjective perception of being powerful within the social context (vs. being low in social power) is related to increased left-sided activity [6, 21, 88]. This effect put together two parts of the same coin: on one side there is the relation between PFC and self-perception, on the other PFC and behavioral performance. In other words, something like a “reinforcing effect” could be hypothesized: from one side the meaning of the joint performance from a social point of view (higher ranking position) seems significant in influencing participants’ outcomes throughout the task (in concomitance with heightened ranking perception and behavioral performance). Here, the modulation of PFC is crucial, thanks to the involvement of social perception processes. On the other side, the improvement in behavioral outcomes could influence self-perceived social position, with subsequent advantages when considering social status. In this case, PFC may be involved to sustain the relation between behavioral outcomes and social representation, thus strenghtening the “social value” of anterior brain networks [89–91].

However, we found also an improved performance in terms of ERs in the case of cooperation compared to competition. That is, we may state that cooperating induces a better perception of self, as well as an increased cognitive performance by the subjects. In other words, it seem probable that the self-perception of well-performing in a cooperative joined-action produces a more consistent and significant cognitive outcome. Based on these results we may suggest that to cooperate is better than to compete also from a cognitive point of view. Previous research showed some contrasting results, since in some cases subjects showed better performances in cognitive tasks during competition than during cooperation [2]. However in that case the absence of a specific “social” feedback during the task and the lacking of a long lasting performance (which might be able to induce the improving of the reciprocal performance across the time) may have partially hidden the consistent differences among the two social conditions.

It should be specified that this increased cognitive and self-perception effect induced by cooperation was mainly remarked by BAS trait. High-BAS participants were better performers and they perceived themselves as higher in ranking. Such effects are consistent with previous findings that proved a left-cortical asymmetry in the case of approach-related motivations, with increased high-frequency electrocortical oscillations over the left with respect to the right PFC [29] and improved self-perception [6, 21]. Such lateralization effect could be explained by considering that the approaching attitude, related to left-sided neural activation, can influence per se both the perceived self-efficacy and competition as well as subjects’ effective outcomes. Thus we could conclude that approach-attitudes and positive emotions could underlie the left-side hyperactivation that reciprocally influences a higher perceived self-efficacy during cooperation, and support a proficient behavioral performance. More generally, high-BAS participants could be more focused on those situations that generate significant positive rewards and proficient, proactive behaviors. These processes are associated with positive affect and self-efficacy to approach social situations [26], as emerged in previous work with analogous conditions [6, 21]. Such BAS trait effect, more related to cooperation than competition, may be due to the fact that individuals with higher-BAS profiles are more active in obtaining their results when a cooperative aim is pursued [92, 93]. By virtue of having relatively a greater proactive attitude, they must rely more on their resources to meet their needs [94].

## Conclusions

To summarize, as reveled by the present results, the contribution of PFC and specifically of left structures is crucial to support the cooperative and competitive joined-action. However, this effect was mainly related with the BAS construct, since the increased left PFC activation for both EEG and fNIRS measures was directly related to the high-BAS trait. Moreover, BAS trait appears to endorse the predisposition to influence self-perceived ranking, as well as actual behavioral performance, since high-BAS participants presented a self-attribution of higher ranking position and general improved cognitive outcomes. Therefore, cooperation proved to be the best condition to perform the task, as underlined by self-perception and cognitive results. In addition, the representation of higher-level in hierarchy related to improved cognitive performance is linked to a clear activity in the DLPFC more for high-BAS people when they operate in a cooperative context.

However, an intrinsic limitation of the present study is related to the low ecological value of the task if compared with the real common situations where cooperation or competition are displayed. Secondly, variations in type of tasks which could not include only a cognitive performance should be provided in future research, to eventually compare the present results with those of more “social” tasks. Thirdly future research should also provide a complete analysis of the inter-personal strategies used by cooperators/competitors by using a more specific hyperscanning methodology in order to consider the joined brain activities of the subjects. Finally the specific cortical side effect and the left/right hemispheric lateralization should be better analyzed also independently from the BAS trait measure.

## Abbreviations

EEG: electroencephalography
fNIRS: functional near-infrared spectroscopy
O2Hb: oxyhemoglobin
ER: error rate
RT: response time
BAS: behavioral activation system
PFC: prefrontal cortex

## References

[1] Munafò MR, Clark T, Flint J (2005). Does measurement instrument moderate the association between the serotonin transporter gene and anxiety-related personality traits? A meta-analysis. Mol Psychiatry.

[2] Funane T, Kiguchi M, Atsumori H, Sato H, Kubota K, Koizumi H (2011). Synchronous activity of two people’s prefrontal cortices during a cooperative task measured by simultaneous near-infrared spectroscopy. J Biomed Opt.

[3] Balconi M, Vanutelli ME (2016). Competition in the brain. The contribution of EEG and fNIRS modulation and personality effects in social ranking. Front Psychol.

[4] Balconi M, Vanutelli ME (2017). Brains in competition. Hyperscanning and cognitive performance in joint-actions. Front Behav Neurosci.

[5] Goldman M, Stockbauer JW, McAuliffe TG (1977). Intergroup and intragroup competition and cooperation. J Exp Soc Psychol.

[6] Balconi M, Pagani S (2015). Social hierarchies and emotions: cortical prefrontal activity, facial feedback (EMG), and cognitive performance in a dynamic interaction. Soc Neurosci.

[7] Cui X, Bryant DM, Reiss AL (2013). NIRS-based hyperscanning reveals increased interpersonal coherence in superior frontal cortex during cooperation. Neuroimage.

[8] Chung D, Yun K, Jeong J (2015). Decoding covert motivations of free riding and cooperation from multi-feature pattern analysis of EEG signals. Soc Cogn Affect Neurosci.

[9] Decety J, Jackson PL, Sommerville JA, Chaminade T, Meltzoff AN (2004). The neural bases of cooperation and competition: an fMRI investigation. Neuroimage.

[10] Liu T, Saito H, Oi M (2015). Role of the right inferior frontal gyrus in turn-based cooperation and competition: a near-infrared spectroscopy study. Brain Cogn.

[11] Decety J, Sommerville JA (2003). Shared representations between self and other: a social cognitive neuroscience view. Trends Cogn Sci.

[12] De Cremer D, Stouten J (2003). When do people find cooperation most justified? The effect of trust and self-other merging in social dilemmas. Soc Justice Res.

[13] Gallagher HL, Frith CD (2003). Functional imaging of “theory of mind”. Trends Cogn Sci.

[14] Leslie A (1987). Pretense and representation: the origins of “‘Theory of Mind’”. Psychol Rev.

[15] Nigg JT (2001). Is ADHD a disinhibitory disorder?. Psychol Bull.

[16] Humphrey NK, Byrne RW, Whiten A (1988). The social function of intellect. Machiavellian intelligence: social expertise and the evolution of intellect in monkeys, apes, humans.

[17] Sebanz N, Knoblich G, Prinz W (2003). Representing others’ actions: just like one’s own?. Cognition.

[18] Knoblich G, Jordan JS (2003). Action coordination in groups and individuals: learning anticipatory control. J Exp Psychol Learn Mem Cogn.

[19] Levitan R, Hasey G, Sloman L, Gilbert P, Sloman L (2000). Major depression and the involuntary defeat strategy: biological correlates. Subordination and defeat: an evolutionary approach to mood disorders and their therapy.

[20] Chiao JY, Adams RBJ, Tse PU, Lowenthal L, Richeson JA, Ambady N (2009). Knowing who’s boss: fMRI and ERP investigations of social dominance perception. Group Process Intergr Relat.

[21] Balconi M, Pagani S (2014). Personality correlates (BAS-BIS), self-perception of social ranking, and cortical (alpha frequency band) modulation in peer-group comparison. Physiol Behav.

[22] Marsh AA, Blair KS, Jones MM, Soliman N, Blair RJR (2009). Dominance and submission: the ventrolateral prefrontal cortex and responses to status cues. J Cogn Neurosci.

[23] Gray JA, Van Goozen SHM, Van de Poll NE, Sergeant JA (1994). Framework for a taxonomy of psychiatric disorder. Emotions: essays on emotion theory.

[24] Demaree HA (2005). Brain lateralization of emotional processing: historical roots and a future incorporating “dominance”. Behav Cogn Neurosci Rev.

[25] Balconi M, Falbo L, Brambilla E (2009). BIS/BAS responses to emotional cues: self report, autonomic measure and alpha band modulation. Pers Individ Differ.

[26] Balconi M, Brambilla E, Falbo L (2009). BIS/BAS, cortical oscillations and coherence in response to emotional cues. Brain Res Bull.

[27] Balconi M, Falbo L, Conte VA (2012). BIS and BAS correlates with psychophysiological and cortical response systems during aversive and appetitive emotional stimuli processing. Motiv Emot.

[28] Balconi M, Mazza G (2009). Brain oscillations and BIS/BAS (behavioral inhibition/activation system) effects on processing masked emotional cues. ERS/ERD and coherence measures of alpha band. Int J Psychophysiol.

[29] Balconi M, Mazza G (2010). Lateralisation effect in comprehension of emotional facial expression: a comparison between EEG alpha band power and behavioural inhibition (BIS) and activation (BAS) systems. Laterality.

[30] Davidson RJ, Ekman P, Saron CD, Senulis JA, Friesen WV (1990). Approach-withdrawal and cerebral asymmetry: emotional expression and brain physiology. I. J Pers Soc Psychol.

[31] Gable SL, Reis HT, Elliot AJ (2000). Behavioral activation and inhibition in everyday life. J Pers Soc Psychol.

[32] Gray JA, McNaughton N (2000). The neuropsychology of anxiety: an enquiry into the functions of the septo-hippocampal system.

[33] Tomarken AJ, Davidson RJ, Wheeler RE, Kinney L (1992). Psychometric properties of resting anterior EEG asymmetry: temporal stability and internal consistency. Psychophysiology.

[34] Seibert LA, Miller JD, Pryor LR, Reidy DE, Zeichner A (2010). Personality and laboratory-based aggression: comparing the predictive power of the five-factor model, BIS/BAS, and impulsivity across context. J Res Pers.

[35] Bechara A, Damasio H, Damasio AR, Lee GP (1999). Different contributions of the human amygdala and ventromedial prefrontal cortex to decision-making. J Neurosci.

[36] Bechara A, Martin EM (2004). Impaired decision making related to working memory deficits in individuals with substance addictions. Neuropsychology.

[37] Sutton SK, Davidson RJ (1997). Prefrontal brain asymmetry: a biological substrate of the behavioral approach and inhibition systems. Psychol Sci.

[38] Balconi M, Vanutelli ME (2015). Emotions and BIS/BAS components affect brain activity (ERPs and fNIRS) in observing intra-species and inter-species interactions. Brain Imaging Behav.

[39] Balconi M, Brambilla E, Falbo L (2009). Appetitive vs. defensive responses to emotional cues. Autonomic measures and brain oscillation modulation. Brain Res.

[40] Balconi M, Bortolotti A, Gonzaga L (2011). Emotional face recognition, EMG response, and medial prefrontal activity in empathic behaviour. Neurosci Res.

[41] Davidson RJ (2004). What does the prefrontal cortex “do” in affect: perspectives on frontal EEG asymmetry research. Biol Psychol.

[42] Harmon-Jones E (2004). On the relationship of frontal brain activity and anger: examining the role of attitude toward anger. Cogn Emot.

[43] Davidson RJ (1992). Emotion and affective style: hemispheric substrates. Psychol Sci.

[44] Knyazev GG (2007). Motivation, emotion, and their inhibitory control mirrored in brain oscillations. Neurosci Biobehav Rev.

[45] Krause L, Enticott PG, Zangen A, Fitzgerald PB (2012). The role of medial prefrontal cortex in theory of mind: a deep rTMS study. Behav Brain Res.

[46] Paré D (2003). Role of the basolateral amygdala in memory consolidation. Prog Neurobiol.

[47] Başar E (1999). Brain function and oscillations: integrative brain function neurophysiology and cognitive processes.

[48] Bekkedal MYV, Rossi J, Panksepp J (2011). Human brain EEG indices of emotions: delineating responses to affective vocalizations by measuring frontal theta event-related synchronization. Neurosci Biobehav Rev.

[49] Karakaş S, Erzengin ÖU, Başar E (2000). The genesis of human event-related responses explained through the theory of oscillatory neural assemblies. Neurosci Lett.

[50] Fernández T, Harmony T, Silva J, Galín L, Díaz-Comas L, Bosch J (1998). Relationship of specific EEG frequencies at specific brain areas with performance. NeuroReport.

[51] Babiloni F, Cincotti F, Mattia D, De Vico Fallani F, Tocci A, Bianchi L, et al. High resolution EEG hyperscanning during a card game. In: 29th Annual international conference on engineering, medicine and biology society, 2007. EMBS 2007. IEEE. 2007;4957–60. 10.1109/IEMBS.2007.4353453 18003119

[52] Elwell CE, Owen-Reece H, Cope M, Wyatt JS, Edwards AD, Delpy DT (1993). Measurement of adult cerebral haemodynamics using near infrared spectroscopy. Acta Neurochir Suppl (Wien).

[53] Balconi M, Grippa E, Vanutelli ME (2015). What hemodynamic (fNIRS), electrophysiological (EEG) and autonomic integrated measures can tell us about emotional processing. Brain Cogn.

[54] Biallas M, Trajkovic I, Haensse D, Marcar V, Wolf M (2012). Reproducibility and sensitivity of detecting brain activity by simultaneous electroencephalography and near-infrared spectroscopy. Exp Brain Res.

[55] Hall JA, Coats EJ, Lebeau LS (2005). Nonverbal behavior and the vertical dimension of social relations: a meta-analysis. Psychol Bull.

[56] Spielberger CD, Gorsuch RL, Lushene RE, Vagg PR, Jacobs GA (1970). STAI manual for the state-trait anxiety inventory.

[57] Beck AT, Steer RA, Brown GK (1996). Manual for the beck depression inventory—II.

[58] Leone L, Pierro A, Mannetti L (2002). Validità della versione italiana delle scale bis/bas di Carver e White (1994): generalizzabilità della struttura e relazioni con costrutti affini. Giornale Italiano di Psicol.

[59] Carver CS, White TL (1994). Behavioral inhibition, behavioral activation, and affective responses to impending reward and punishment: the BIS/BAS Scales. J Pers Soc Psychol.

[60] Oostenveld R, Praamstra P (2001). The five percent electrode system for high-resolution EEG and ERP measurements. Clin Neurophysiol.

[61] Ludwig KA, Miriani RM, Langhals NB, Joseph MD, Anderson DJ, Kipke DR (2009). Using a common average reference to improve cortical neuron recordings from microelectrode arrays. J Neurophysiol.

[62] Sapolsky RM (2004). Social status and health in humans and other animals. Annu Rev Anthropol.

[63] Pfurtscheller G (1992). Event-related synchronization (ERS): an electrophysiological correlate of cortical areas at rest. Electroencephalogr Clin Neurophysiol.

[64] Chung D, Yun K, Jeong J. Neural mechanisms of free-riding and cooperation in a public goods game: an EEG hyperscanning study. In: Proceedings of 6th international conference on cognitive science 2008. p. 2–5.

[65] Matsuda G, Hiraki K (2006). Sustained decrease in oxygenated hemoglobin during video games in the dorsal prefrontal cortex: a NIRS study of children. Neuroimage.

[66] Schroeter ML, Zysset S, Kruggel F, Von Cramon DY (2003). Age dependency of the hemodynamic response as measured by functional near-infrared spectroscopy. Neuroimage.

[67] Shimada S, Hiraki K (2006). Infant’s brain responses to live and televised action. Neuroimage.

[68] Karafin MS, Tranel D, Adolphs R (2004). Dominance attributions following damage to the ventromedial prefrontal cortex. J Cogn Neurosci.

[69] Suzuki S, Niki K, Fujisaki S, Akiyama E (2011). Neural basis of conditional cooperation. Soc Cogn Affect Neurosci.

[70] Haruno M, Kawato M (2009). Activity in the superior temporal sulcus highlights learning competence in an interaction game. J Neurosci.

[71] De Vico Fallani F, Nicosia V, Sinatra R, Astolfi L, Cincotti F, Mattia D (2010). Defecting or not defecting: how to “read” human behavior during cooperative games by EEG measurements. PLoS ONE.

[72] Harmon-Jones E, Allen JJB (1998). Anger and frontal brain activity: EEG asymmetry consistent with approach motivation despite negative affective valence. J Pers Soc Psychol.

[73] Balconi M, Vanutelli ME (2016). Vocal and visual stimulation, congruence and lateralization affect brain oscillations in interspecies emotional positive and negative interactions. Soc Neurosci.

[74] Kawasaki M, Yamaguchi Y (2013). Frontal theta and beta synchronizations for monetary reward increase visual working memory capacity. Soc Cogn Affect Neurosci.

[75] Moore RA, Gale A, Morris PH, Forrester D (2006). Theta phase locking across the neocortex reflects cortico-hippocampal recursive communication during goal conflict resolution. Int J Psychophysiol.

[76] Neo PS, Thurlow JK, McNaughton N (2011). Stopping, goal-conflict, trait anxiety and frontal rhythmic power in the stop signal task. Cogn Affect Behav Neurosci.

[77] Savostyanov AN, Tsai AC, Liou M, Levin EA, Juin-Der L, Yuryganov AV (2009). EEG correlates of trait anxiety in the stop-signal paradigm. Neurosci Lett.

[78] Yun K, Watanabe K, Shimojo S (2012). Interpersonal body and neural synchronization as a marker of implicit social interaction. Sci Rep.

[79] Koslow K, Mendes WB, Pajtas PE, Pizzagalli DA (2013). Greater left resting intracortical activity as a buffer to social threat. Psychol Sci.

[80] Barron B (2003). When smart groups fail. J Learn Sci.

[81] Elliott R, Frith CD, Dolan RJ (1997). Differential neural response to positive and negative feedback in planning and guessing tasks. Neuropsychologia.

[82] Elliott R, Newman JL, Longe OA, Deakin JFW (2003). Differential response patterns in the striatum and orbitofrontal cortex to financial reward in humans: a parametric functional magnetic resonance imaging study. J Neurosci.

[83] Tremblay L, Schultz W (1999). Relative reward preference in primate orbitofrontal cortex. Nature.

[84] Balconi M, Finocchiaro R, Canavesio Y (2015). Left hemispheric imbalance and reward mechanisms affect gambling behavior: the contribution of the metacognition and cortical brain oscillations. Clin EEG Neurosci.

[85] Balconi M, Finocchiaro R (2016). Deficit in rewarding mechanisms and prefrontal left/right cortical effect in vulnerability for internet addiction. Acta Neuropsychiatr.

[86] Davidson RJ, Davidson RJ, Hughdahl K (1995). Cerebral asymmetry, emotion and affective style. Brain asymmetry.

[87] Elliott R, Dolan RJ, Frith CD (2000). Dissociable functions in the medial and lateral orbitofrontal cortex: evidence from human neuroimaging studies. Cereb Cortex.

[88] Boksem MAS, Smolders R, Cremer DD (2012). Social power and approach-related neural activity. Soc Cogn Affect Neurosci.

[89] Balconi M, Canavesio Y (2014). High-frequency rTMS on DLPFC increases prosocial attitude in case of decision to support people. Soc Neurosci.

[90] Lev-Ran S, Shamay-Tsoory SG, Zangen A, Levkovitz Y (2012). Transcranial magnetic stimulation of the ventromedial prefrontal cortex impairs theory of mind learning. Eur Psychiatry.

[91] Wang J, Wang Y, Hu Z, Li X (2014). Transcranial direct current stimulation of the dorsolateral prefrontal cortex increased pain empathy. Neuroscience.

[92] Magee JC, Galinsky AD (2008). Social hierarchy: the self-reinforcing nature of power and status. Acad Manag Ann.

[93] Pothos EM, Busemeyer JR (2009). A quantum probability explanation for violations of “rational” decision theory. Proc R Soc B Biol Sci.

[94] Kraus MW, Piff PK, Keltner D (2009). Social class, sense of control, and social explanation. J Pers Soc Psychol.

